# Risk of developing chronic kidney disease in young-onset Type 2 diabetes in Korea

**DOI:** 10.1038/s41598-023-36711-2

**Published:** 2023-06-21

**Authors:** Joonyub Lee, Seung-Hwan Lee, Kun-Ho Yoon, Jae Hyoung Cho, Kyungdo Han, Yeoree Yang

**Affiliations:** 1 Division of Endocrinology and Metabolism, Department of Internal Medicine, Seoul St. Mary’s Hospital, College of Medicine, The Catholic University of Korea, Seoul, Korea; 2 Department of Medical Informatics, College of Medicine, The Catholic University of Korea, 222, Banpo-Daero, Seocho-Gu, Seoul, 06591 Republic of Korea; 3 Catholic Smart Health Care Center, The Catholic University of Korea, Seoul, Korea; 4 Department of Statistics and Actuarial Science, Soongsil University, 369 Sangdo-Ro, Dongjak-Gu, Seoul, 06978 Korea

**Keywords:** Epidemiology, Endocrine system and metabolic diseases

## Abstract

We investigated the risk of developing chronic kidney disease (CKD) in patients with young-onset Type 2 diabetes (YOD, diagnosed age < 40 years). We enrolled 84,384 patients aged 20–64 who started anti-diabetic medication between 2010 and 2011 from the Korea National Health Insurance Sharing Service; patients with Type 1 diabetes or a history of CKD were excluded. Multivariate logistic regression analyses were performed to adjust for YOD-distinct variables and compare the incidence of CKD between YOD and late-onset diabetes (LOD, diagnosed age ≥ 40 years). During the median observation period of 5.16 years (interquartile range: 4.58–5.77 years), 1480 out of 77,039 LOD patients and 34 out of 7345 YOD patients developed CKD. Patients with YOD had distinct baseline characteristics compared with the patients with LOD. The odds ratio of developing CKD in patients with YOD over LOD was 1.70 (95% CI 1.15–2.51) after adjusting clinically distinct variables. The increased CKD odds in YOD compared with LOD was greater in the non-smoking group (OR 2.03, 95% CI 1.26–3.26) than in the smoking group (OR 1.49, 95% CI 0.74–2.98, p = 0.0393 for interaction). Among YOD patients, hypertension (34.76% vs. 64.71%, p = 0.0003), dyslipidemia (46.87% vs. 73.53%, p = 0.0019), and sulfonylurea use (35.54% vs. 52.94%, p = 0.0345) were associated with CKD development. YOD patients have a greater risk of developing CKD than LOD patients after adjusting clinically distinct variables.

## Introduction

Type 2 diabetes mellitus (T2DM) is a complex metabolic disorder that increases the risk for vascular complications. Although T2DM commonly develops in mid-to-old age (> 40 years), the number of patients who develop T2DM at a young age (< 40 years) is increasing^1^. The prevalence of T2DM in young populations varies widely among ethnicities and can reach an estimated 5.3%^2–4^. Especially in Asia, the incidence of young-onset T2DM (YOD, diagnosed age < 40 years) is increasing rapidly. The prevalence of T2DM in Chinese adolescents was reported in 2010 to be more than double that in 1995^5^. The incidence of T2DM in children in Thailand increased more than threefold from 1997–1999 compared with 1987–1996^6^.

Patients with YOD are reported to have clinical characteristics that are distinct from those of patients with late-onset diabetes (LOD). Patients with YOD are reported to be more obese, have poor adherence to medical treatment, have a more rapid decline in β-cell function, and use insulin earlier after diagnosis than patients with LOD^4,7–11^. These characteristics suggest that patients with YOD may have different pathophysiology and follow different clinical paths than patients with LOD. Indeed, previous studies have reported that patients with YOD are at an increased risk of mortality and macrovascular complications compared with patients with LOD^12–14^. However, whether patients with YOD are at an increased risk of developing microvascular diseases compared with those with LOD remains inconclusive. Previous studies have reported conflicting results regarding the incidence of retinopathy in patients with YOD and LOD^15,16^. For nephropathy, which is one of the most common diabetic complications that cause substantial medical and socioeconomic burdens, the relative risk in YOD compared with LOD remains unclear^7^.

Diabetic nephropathy can be delayed or prevented using intensive metabolic (glucose and blood pressure) management^17–19^. Therefore, understanding the risk of developing renal complications in T2DM subgroups is important for determining on whom limited medical resources should be focused. In this study, we explored the risk of nephropathy in patients with YOD and LOD by retrospectively analyzing a large number of Korean patients with T2DM.

## Subjects

### Data source and study population

**Figure 1:**
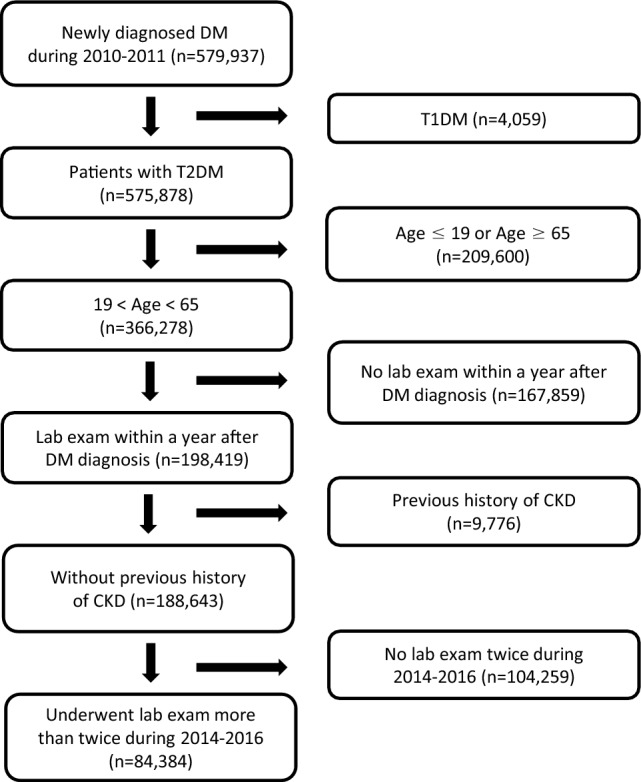
Study scheme. Patients newly diagnosed 579,937 with diabetes were enrolled in this study. A total of 84,384 (YOD = 7345, LOD = 77,039) patients were analyzed to estimate the risk of CKD development. *CKD* chronic kidney disease, *DM* diabetes mellitus, *LOD* late-onset diabetes, *T1DM* Type 1 DM, *T2DM* Type 2 DM, *YOD* young-onset Type 2 diabetes.

This was a longitudinal retrospective observational study using a customized National Health Information database from the National Health Insurance Sharing Service^20^. The National Health Insurance Service (NHIS), a single insurer managed by the government, administers a mandatory universal insurance system for all citizens who reside in South Korea. It provides regular health check-up programs for all citizens older than 20 years, at least biennially. Since 2014, the NHIS has released nationally representative sample databases that include nearly the entire Korean population and are open to all researchers whose study protocols are approved by an official review committee. The database comprises four sections: participants’ insurance eligibility database (e.g., age, sex, socioeconomic variables, type of eligibility, and income level), a medical procedure and treatment database (based on the medical bills that were claimed by medical service providers for their medical expense claims), a health examination database (results of general health examinations and questionnaires on lifestyle and behavior), and a medical care institution database (types of medical care institutions, location, equipment, and the number of physicians)^21^. The diagnosis of Type 2 diabetes was based on operation definition which requires related ICD-10 codes (E11–14) and prescription code of anti-diabetic medications. We included 579,937 subjects who had their first claim for the prescription of anti-diabetic medications between January 1, 2010, and December 31, 2011 (index year), with at least one claim per year under diabetes-related International Classification of Disease, 10th Revision (ICD-10) codes E11–14. For patients with type 1 diabetes are known to exhibit different clinical course of renal complications from those with Type 2 diabetes in young population, subjects with type 1 diabetes (ICD-10 code: E10) were not included^22,23^. Among the subjects, we excluded those who were aged < 20 or > 65 years, those who did not have health examination data within 1 year of the index year, and those with a previous history of overt chronic kidney disease (CKD) before the index year, as indicated by a baseline glomerular filtration rate (GFR) calculated by the Modification of Diet in Renal Disease (MDRD) equation of < 60 mL/min/1.73 m^2^, the presence of CKD-related ICD-10 codes, or a history of renal replacement therapy in the claims database. Ultimately, we analyzed 84,384 subjects who underwent a health examination at least two times between 2014 and 2016 (Fig. 1).

## Materials and methods

### Definitions and measurements

Among enrolled subjects, YOD was defined as subjects who started anti-diabetic medication and had their first claim for the prescription at an age of < 40 years. Thus, the YOD data of patients between the ages of 20 and < 40 years was compared with data from subjects classified as LOD, who started anti-diabetic medication between the ages of 40 and 65 years. To minimize non-diabetes-related CKD, subjects aged over 65 years were excluded for elderly onset CKD is known to have heterogeneous etiology^24,25^.

Body mass index (BMI) was calculated as the subject’s weight in kilograms divided by the square of the subject’s height in meters; obesity was defined as a BMI ≥ 25 kg/m^2^. Abdominal obesity was defined as a waist circumference (WC) > 90 cm for men and > 85 cm for women (using the modified WC criteria of the Korean Society for the Study of Obesity)^26^. The presence of hypertension was defined as the presence of at least one claim per year under ICD-10 codes I10-13 or I15 and at least one claim per year for the prescription of antihypertensive agents or a systolic/diastolic blood pressure ≥ 140/90 mmHg. The presence of dyslipidemia was defined as the presence of at least one claim per year under ICD-10 code E78 and at least one claim per year for the prescription of lipid-lowering agents or total cholesterol ≥ 240 mg/dL.

Information on the participants’ current smoking status, alcohol consumption, and degree of exercise was obtained using a questionnaire from the health examination database; alcohol intake was categorized into < 30 g/day and ≥ 30 g/day; regular exercise was defined as the performance of > 20 min of strenuous physical activity at least three times per week or > 30 min of moderate physical activity at least five times per week. Household income level was dichotomized at the lower 20% of those receiving medical aid. Blood samples for the measurement of blood glucose and serum cholesterol levels were obtained after overnight fasting.

### Study outcomes and follow-up

The primary endpoint of the study was the new development of CKD, which was defined as a GFR of < 60 mL/min/1.73 m^2^ measured consecutively twice or more (stage 3 or higher) during the health examination between January 1, 2014, and December 31, 2016. The GFR was calculated using the MDRD equation: GFR = 186 × (serum creatinine)^−1.154^ × (age)^−0.203^ × 0.742 (if female).

### Statistical analysis

The baseline characteristics of the participants are presented as mean ± standard deviation, median (interquartile range), or n (%). The demographic and clinical differences between the YOD and LOD groups of continuous variables were evaluated using a t-test; categorical variables were analyzed using a χ^2^ test. Multivariable logistic regression analysis was performed to evaluate the association between two groups (YOD vs. LOD) and the risk of CKD development. Odds ratios (OR) and 95% confidence intervals (CI) were calculated after adjusting for potential confounders. Model 1 was adjusted for age, sex, and BMI. Model 2 was further adjusted for smoking, heavy drinking, regular exercise, and low-income status. Model 3 was further adjusted for the presence of hypertension, dyslipidemia, and aspirin use. Model 4 was further adjusted for fasting glucose levels, insulin use, and the number of oral hypoglycemic agents. Subgroup analysis was performed according to sex, smoking status, heavy drinking, regular exercise, low income, comorbidities, (including obesity, hyperlipidemia, and hypertension), medication status (including use of aspirin, angiotensin II receptor blocker, statin, and insulin), and the number of oral hypoglycemic agents. Statistical analyses were performed using SAS version 9.4 (SAS Institute Inc., Cary, NC, USA). A 2-tailed p-value < 0.05 was considered statistically significant.

### Ethics approval

This study was approved by the Institutional Review Board of the Catholic University of Korea (No. KC20ZASI0271). The need for informed consent was waived by Institutional Review Board of the Catholic University of Korea because anonymous and de-identified information was used for the analyses. All procedures related to human participants were performed in accordance with the ethical standards of the Helsinki Declaration.

## Results

### Clinical characteristics of the study participants

This was a longitudinal retrospective observational study with a median observational period of 5.16 years (interquartile range: 4.58–5.77 years). Of 579,937 patients enrolled initially, a final number of 84,384 (YOD = 7345; LOD = 77,039) patients newly diagnosed with T2DM were analyzed. The detailed enrollment criteria are described in the *Subjects* section above. The average age of patients with YOD was 35.06 ± 3.64 years; the LOD group averaged 52.9 ± 6.52 years old. In agreement with previous reports, patients with YOD had clinical characteristics distinct from those of patients with LOD^10,11,27–30^. The patients with YOD had a higher proportion of males (YOD 81.59% vs. LOD 63.37%, p < 0.0001), current smokers (YOD 45.8% vs. LOD 26.86%, p < 0.0001), and heavy drinkers (YOD 12.89% vs. LOD 10.79%, p < 0.0001) than the patients with LOD. On the contrary, the YOD group had a lower proportion of patients with regular physical activity (YOD 16.86% vs. LOD 21.62%, p < 0.0001) and low socioeconomic income (13.08% vs. 22.56%, p < 0.0001) than the LOD group. The patients with YOD had a lower proportion of hypertension and angiotensin II receptor–blocker use; the systolic blood pressure was lower, while the diastolic blood pressure was comparable to that in patients with LOD. The YOD group had a lower proportion of dyslipidemia, statin users, aspirin users, and patients with low levels of high-density lipoprotein cholesterol, but had higher average levels of total cholesterol, triglycerides, and low-density lipoprotein cholesterol than patients with LOD. Moreover, the patients with YOD had a higher average BMI (YOD 25.47 vs. LOD 26.98 kg/m^2^, p < 0.0001), waist circumference, and fasting plasma glucose level (YOD 158.79 vs. LOD 143.36 mg/dL, p < 0.0001), higher proportion of insulin users (YOD 12.13% vs. LOD 6.69%, p < 0.0001), and used a greater number of oral hypoglycemic agents (YOD 1.47% vs. LOD 1.35%, p < 0.0001) than the patients with LOD. Importantly, the patients with YOD had a higher baseline GFR (YOD 98.92 vs. LOD 91.33 mL/min/1.73 m^2^, p < 0.0001) compared with the patients with LOD (Table 1).

**Table 1:** Baseline characteristics of subjects.

	LOD	YOD	p-value
Number	77,039	7345	
Sex: male, n (%)	48,973 (63.57)	5993 (81.59)	< 0.0001
Age	52.9 ± 6.52	35.06 ± 3.64	< 0.0001
Age, n (%)			< 0.0001
20–39	0 (0)	7345 (100)	
40 s	23,972 (31.12)	0 (0)	
50 s	38,535 (50.02)	0 (0)	
60–64	14,532 (18.86)	0 (0)	
Height (cm)	163.83 ± 8.54	170.58 ± 7.69	< 0.0001
Weight (kg)	68.56 ± 11.14	78.77 ± 14.45	< 0.0001
BMI (kg/m^2^)	25.47 ± 3.21	26.98 ± 4.16	< 0.0001
Obesity (BMI ≥ 25 kg/m^2^), n (%)	40,857 (53.03)	4974 (67.72)	< 0.0001
Waist circumference (cm)	85.65 ± 8.3	88.16 ± 10.15	< 0.0001
Abdominal obesity, n (%)	60,227 (78.18)	6006 (81.77)	< 0.0001
Systolic BP (mmHg)	127.31 ± 14.87	125.88 ± 14.01	< 0.0001
Diastolic BP (mmHg)	79.73 ± 9.96	79.84 ± 10.17	0.3418
Smoking, n (%)	20,693 (26.86)	3364 (45.8)	< 0.0001
Heavy drinker, n (%)	8312 (10.79)	947 (12.89)	< .0001
Regular physical activity, n (%)	16,659 (21.62)	1238 (16.86)	< 0.0001
Low socioeconomic status, n (%)	17,382 (22.56)	961 (13.08)	< 0.0001
Hypertension, n (%)	41,885 (54.37)	2563 (34.89)	< 0.0001
Dyslipidemia, n (%)	40,144 (52.11)	3452 (47)	< 0.0001
Medication, n (%)			
Aspirin	21,521 (27.94)	753 (10.25)	< 0.0001
Statin	36,971 (47.99)	2784 (37.9)	< 0.0001
ARB	29,655 (38.49)	1795 (24.44)	< 0.0001
Insulin, n (%)	5157 (6.69)	891 (12.13)	< 0.0001
Oral hypoglycemic agents, n (%)			
Sulfonylurea	25,888 (33.6)	2616 (35.62)	0.0005
Metformin	60,640 (78.71)	5903 (80.37)	0.0009
Meglitinide	678 (0.88)	64 (0.87)	0.9389
Thiazolidinedione	2209 (2.87)	228 (3.1)	0.247
DPP-4 inhibitor	6482 (8.41)	783 (10.66)	< 0.0001
α-Glucosidase inhibitor	2759 (3.58)	288 (3.92)	0.1359
Number of oral hypoglycemic agents	1.35 ± 0.54	1.47 ± 0.6	< 0.0001
Number of oral hypoglycemic agents			< 0.0001
1	52,760 (68.48)	4295 (58.48)	
2	21,981 (28.53)	2713 (36.94)	
≥ 3	2298 (2.98)	337 (4.59)	
Fasting glucose (mg/dl)	143.36 ± 49.34	158.79 ± 65.87	< 0.0001
GFR (ml/min/1.73 m^2^)	91.33 ± 36.64	98.92 ± 45.06	< 0.0001
Total cholesterol (mg/dl)	203.46 ± 42.27	205.54 ± 42.67	< 0.0001
Triglyceride (mg/dl)	153.07 (152.47–153.68)	178.55 (176.04–181.1)	< 0.0001
HDL-C (mg/dl)	51.18 ± 14.54	48.58 ± 15.31	< 0.0001
LDL-C (mg/dl)	117.4 2 ± 45.43	116.38 ± 53.41	0.0663

All data was expressed as mean ± standard deviation, median (interquartile range), median (95% CI), or n (%). Student’s t-test (continuous variables) and χ^2^ test (categorical variables) was used for statistical analysis and p-value < 0.05 was regarded as statistical significance. The baseline characteristics between patients with YOD and LOD were compared.

*ARB* angiotensin II receptor blocker, *BMI* Body mass index, *DBP* Diastolic blood pressure, *DPP-4 inhibitor* dipeptidyl peptidase 4, *GFR* Glomerular Filtration Rate, *HDL-C* High density lipoprotein cholesterol, *LDL-C* Low density lipoprotein cholesterol, *LOD* late-onset diabetes; *SBP* Systolic blood pressure, *YOD* young-onset Type 2 diabetes.

### YOD patients are more prone to develop CKD

During the observational period, 1442 patients (34 YOD and 1408 LOD) developed CKD (Table 2). Because the purpose of this study was to examine whether patients with LOD and YOD are fundamentally different in terms of the risk of developing CKD, and YOD subjects were different from LOD subjects in several clinical variables (Table 1), we compared the risk of developing CKD in YOD and LOD subjects after serially adjusting for clinically distinct variables (Table 2). When age, sex, and BMI were adjusted for (Model 1), the OR of developing CKD in YOD was 1.70 (95% CI 1.15–2.52) compared with LOD. When smoking, heavy drinking, and physical activity were further adjusted for (Model 2), the OR of developing CKD in YOD was 1.69 (95% CI 1.14–2.50) compared with LOD. The OR of developing CKD in YOD compared with LOD was 1.71 (95% CI 1.15–2.52) when hypertension, dyslipidemia, and aspirin were further adjusted for (Model 3). Ultimately, YOD patients showed an increased risk of developing CKD when the level of fasting glucose, insulin use, and the number of oral hypoglycemic agents, were additionally adjusted for in Model 4 (OR 1.70, 95% CI 1.15–2.51).

**Table 2:** The risk of developing CKD in patients with YOD and LOD after adjusting clinically distinct variables.

Type	n	CKD developed	Odds ratio (95% CI)			
			Model 1	Model 2	Model 3	Model 4
LOD	77,039	1480	1 (ref.)	1 (ref.)	1 (ref.)	1 (ref.)
YOD	7345	34	1.70 (1.15, 2.52)	1.69 (1.14, 2.50)	1.71 (1.15, 2.52)	1.70 (1.15, 2.51)

Odds ratios of CKD development in LOD and YOD are expressed after adjusting variables by multivariate logistic regression analysis. Model 1 was adjusted for age, sex, BMI. Model 2 was adjusted for Model 1 + smoking, heavy drinking, regular physical activity, low socioeconomic status. Model 3 was adjusted for Model 2 + hypertension, dyslipidemia, aspirin. Model 4 was adjusted for Model 3 + fasting glucose, Insulin, number of oral hypoglycemic agents.

*CKD* chronic kidney disease, *LOD* late-onset diabetes, *YOD* young-onset Type 2 diabetes;

### Relationship between the clinical characteristics of participants and the development of CKD

We performed further subgroup analyses to identify which patient characteristics were associated with a high risk of developing CKD in patients with YOD compared with patients with LOD. The increased incidence of CKD in YOD was greater in the non-smoking group (OR 2.03, 95% CI 1.26–3.26) than in the smoking group (OR 1.49, 95% CI 0.74–2.98, p = 0.0393 for interaction) (Table 3). Patients with low BMI (OR 2.48, 95% CI 1.37–4.51) were also associated with a higher incidence of CKD development in YOD compared with patients with high BMI (OR 1.46, 95% CI 0.87–2.45, p = 0.0145 for interaction) (Table 3). There was no significant difference in risk of developing CKD between non-ARB users (HR 1.36, 95% CI 0.75–2.46)) and ARB users (HR 2.16, 95% CI 1.28–3.65, p for interaction: 0.25) (Table 3).

**Table 3:** Risk of CKD in YOD compared with LOD by subgroups.

Subgroup	Type	n	CKD (n)	CKD (%)	Odds ratio (95% CI)				p for inter-action
					Model 1	Model 2	Model 3	Model 4	
Male	LOD	48,973	935	1.91	1 (ref.)	0.92	1 (ref.)	1 (ref.)	0.9183
	YOD	5993	30	0.5	1.48 (0.97, 2.25)	1.44 (0.94, 2.195)	1.47 (0.96, 2.24)	1.46 (0.96, 2.24)	
Female	LOD	28,066	473	1.69	1 (ref.)	1 (ref.)	1 (ref.)	1 (ref.)	
	YOD	1352	4	0.3	2.52 (0.87, 7.32)	2.54 (0.87, 7.39)	2.66 (0.91, 7.76)	2.665 (0.91, 7.77)	
Non-smoker	LOD	56,346	1047	1.86	1 (ref.)	0.04	1 (ref.)	1 (ref.)	0.0393
	YOD	3981	23	0.58	2.03 (1.27, 3.27)	2.01 (1.25, 3.23)	2.05 (1.27, 3.29)	2.03 (1.26, 3.26)	
Current smoker	LOD	20,693	361	1.74	1 (ref.)	1 (ref.)	1 (ref.)	1 (ref.)	
	YOD	3364	11	0.33	1.47 (0.73, 2.93)	1.45 (0.72, 2.89)	1.469 (0.73, 2.94)	1.49 (0.74, 2.98)	
Non-heavy drinker	LOD	68,727	1284	1.87	1 (ref.)	0.48	1 (ref.)	1 (ref.)	0.482
	YOD	6398	31	0.48	1.89 (1.25, 2.85)	1.89 (1.25, 2.85)	1.92 (1.27, 2.90)	1.913 (1.27, 2.89)	
Heavy drinker	LOD	8,312	124	1.49	1 (ref.)	1 (ref.)	1 (ref.)	1 (ref.)	
	YOD	947	3	0.32	0.69 (0.20, 2.44)	0.69 (0.20, 2.42)	0.68 (0.19, 2.38)	0.67 (0.19, 2.36)	
Non-regular physical activity	LOD	60,380	1091	1.81	1 (ref.)	0.80	1 (ref.)	1 (ref.)	0.7967
	YOD	6107	29	0.47	1.69 (1.11, 2.60)	1.68 (1.10, 2.58)	1.70 (1.11, 2.61)	1.69 (1.10, 2.59)	
Regular physical activity	LOD	16,659	317	1.9	1 (ref.)	1 (ref.)	1 (ref.)	1 (ref.)	
	YOD	1238	5	0.4	1.69 (0.63, 4.52)	1.63 (0.61, 4.36)	1.63 (0.61, 4.37)	1.67 (0.62, 4.47)	
Non-low socioeconomic status	LOD	59,657	1013	1.7	1 (ref.)	0.36	1 (ref.)	1 (ref.)	0.3627
	YOD	6384	27	0.42	1.55 (0.10, 2.41)	1.53 (0.99, 2.38)	1.56 (1.00, 2.42)	1.54 (0.99, 2.40)	
Low socioeconomic status	LOD	17,382	395	2.27	1 (ref.)	1 (ref.)	1 (ref.)	1 (ref.)	
	YOD	961	7	0.73	2.48 (1.06, 5.82)	2.46 (1.05, 5.77)	2.46 (1.05, 5.76)	2.46 (1.05, 5.77)	
BMI < 25 kg/m^2^	LOD	36,182	587	1.62	1 (ref.)	0.01	1 (ref.)	1 (ref.)	0.0145
	YOD	2371	15	0.63	2.59 (1.43, 4.70)	2.56 (1.41, 4.65)	2.52 (1.39, 4.57)	2.49 (1.37, 4.52)	
BMI ≥ 25 kg/m^2^	LOD	40,857	821	2.01	1 (ref.)	1 (ref.)	1 (ref.)	1 (ref.)	
	YOD	4974	19	0.38	1.41 (0.84, 2.36)	1.39 (0.83, 2.34)	1.44 (0.85, 2.41)	1.43 (0.85,2.41)	
Non-hypertension	LOD	35,154	330	0.94	1 (ref.)	0.34	1 (ref.)	1 (ref.)	0.3446
	YOD	4782	12	0.25	1.62 (0.82, 3.19)	1.60 (0.81, 3.15)	1.61 (0.82, 3.17)	1.60 (0.81, 3.15)	
Hypertension	LOD	41,885	1078	2.57	1 (ref.)	1 (ref.)	1 (ref.)	1 (ref.)	
	YOD	2563	22	0.86	1.83 (1.13, 2.97)	1.81 (1.12, 2.93)	1.83 (1.13, 2.96)	1.83 (1.13, 2.96)	
Non-dyslipidemia	LOD	36,895	547	1.48	1 (ref.)	0.10	1 (ref.)	1 (ref.)	0.1032
	YOD	3893	9	0.23	1.00 (0.49, 2.07)	0.99 (0.48, 2.04)	1.01 (0.49, 2.09)	1.00 (0.49, 2.08)	
Dyslipidemia	LOD	40,144	861	2.14	1 (ref.)	1 (ref.)	1 (ref.)	1 (ref.)	
	YOD	3452	25	0.72	2.28 (1.43, 3.66)	2.26 (1.41, 3.62)	2.25 (1.41, 3.61)	2.25 (1.40, 3.60)	
Non-aspirin	LOD	55,518	797	1.44	1 (ref.)	0.63	1 (ref.)	1 (ref.)	0.6295
	YOD	6592	29	0.44	1.91 (1.23, 2.97)	1.89 (1.22, 2.95)	1.88 (1.21, 2.92)	1.88 (1.21, 2.93)	
Aspirin	LOD	21,521	611	2.84	1 (ref.)	1 (ref.)	1 (ref.)	1 (ref.)	
	YOD	753	5	0.66	1.28 (0.50, 3.26)	1.26 (0.49, 3.22)	1.27 (0.50, 3.24)	1.26 (0.50, 3.23)	
Non ARB	LOD	47,384	552	1.16	1 (ref.)	0.25	1 (ref.)	1 (ref.)	0.2454
	YOD	5550	15	0.27	1.44 (0.80, 2.61)	1.43 (0.79, 2.58)	1.380 (0.76, 2.49)	1.36 (0.75, 2.46)	
ARB	LOD	29,655	856	2.89	1 (ref.)	1 (ref.)	1 (ref.)	1 (ref.)	
	YOD	1795	19	1.06	2.18 (1.29, 3.68)	2.15 (1.27, 3.62)	2.14 (1.27, 3.61)	2.16 (1.28, 3.65)	
Non-statin	LOD	40,068	581	1.45	1 (ref.)	0.05	1 (ref.)	1 (ref.)	0.0519
	YOD	4561	11	0.24	1.02 (0.53,1.97)	1.01 (0.52,1.96)	1.02 (0.53, 1.97)	1.01 (0.52, 1.96)	
Statin	LOD	36,971	827	2.24	1 (ref.)	1 (ref.)	1 (ref.)	1 (ref.)	
	YOD	2784	23	0.83	2.48 (1.52, 4.05)	2.44 (1.50, 3.99)	2.48 (1.52, 4.05)	2.47 (1.51, 4.04)	
Non-insulin	LOD	71,882	1294	1.8	1 (ref.)	0.67	1 (ref.)	1 (ref.)	0.6704
	YOD	6454	29	0.45	1.67 (1.10, 2.54)	1.65 (1.09, 2.52)	1.66 (1.09, 2.53)	1.67 (1.10, 2.54)	
Insulin	LOD	5157	114	2.21	1 (ref.)	1 (ref.)	1 (ref.)	1 (ref.)	
	YOD	891	5	0.56	1.59 (0.53, 4.83)	1.57 (0.52, 4.76)	1.71 (0.56, 5.23)	1.71 (0.56, 5.23)	
Number of oral hypoglycemic agents < 3	LOD	74,741	1360	1.82	1 (ref.)	0.46	1 (ref.)	1 (ref.)	0.4642
	YOD	7008	31	0.44	1.65 (1.10, 2.48)	1.63 (1.09, 2.45)	1.65 (1.10, 2.48)	1.65 (1.10, 2.48)	
Number of oral hypoglycemic agents ≥ 3	LOD	2298	48	2.09	1 (ref.)	1 (ref.)	1 (ref.)	1 (ref.)	
	YOD	337	3	0.89	2.44 (0.54, 11.07)	2.45 (0.54, 11.15)	2.42 (0.53, 11.06)	2.45 (0.54, 11.23)	

Odds ratios of CKD development in LOD and YOD are expressed after adjusting variables by multivariate logistic regression analysis by subgroups. Model 1 was adjusted for age, sex, BMI. Model 2 was adjusted for Model 1 + smoking, heavy drinking, regular physical activity, low socioeconomic status. Model 3 was adjusted for Model 2 + hypertension, dyslipidemia, aspirin. Model 4 was adjusted for Model 3 + fasting glucose, Insulin, number of oral hypoglycemic agents. *ARB* angiotensin II receptor blocker, *BMI* Body mass index, *CKD* chronic kidney disease, *DBP* Diastolic blood pressure, *GFR* Glomerular Filtration Rate, *HDL-C* High density lipoprotein cholesterol, *LDL-C* Low density lipoprotein cholesterol, *LOD* late-onset diabetes, *SBP* Systolic blood pressure, *YOD* young-onset Type 2 diabetes.

We further analyzed the clinical characteristics of patients with YOD who developed CKD. Among the YOD group, patients who developed CKD were associated with a high prevalence of hypertension (34.76% vs. 64.71%, p = 0.0003), dyslipidemia (46.87% vs. 73.53%, p = 0.0019), and sulfonylurea use (35.54% vs. 52.94%, p = 0.0345) (Suppl. Table 1). In summary, patients with YOD had an increased risk of developing CKD compared with patients with LOD after adjusting for clinically distinct variables. Non-smoking or low-BMI patients who develop T2DM at an early age are associated with a high incidence of CKD. Among YOD patients, those who developed CKD were associated with a high prevalence of concomitant hypertension or dyslipidemia and the use of sulfonylurea.

## Discussion

In this study, we retrospectively analyzed patients with newly diagnosed T2DM in Korea. Our study demonstrated that patients with YOD are at a higher risk of developing CKD than those with LOD after adjusting for their clinically distinct characteristics. Patients with YOD were characterized by poor metabolic risk factors such as high BMI, high fasting glucose levels, smoking, heavy drinking, and low physical activity; however, they shared some favorable metabolic characteristics, including a lower prevalence of hypertension, dyslipidemia, and low income. The risk of developing CKD was consistently higher in patients with YOD than in those with LOD (OR: 1.7) when clinically distinct variables were adjusted (Table 2). The higher risk of CKD development in patients with YOD was consistent in the subgroup analysis and was especially prominent in non-smoking patients with a low BMI (Table 3).

Our results suggest that patients with YOD are an important subpopulation of T2DM cases, with respect to their renal outcome. Diabetic nephropathy is a progressive disorder that may result in end-stage renal disease (ESRD), in which patients require renal replacement therapy. Patients with CKD are also increased in risk of mortality, fluid retention, uncontrolled blood pressure, electrolyte imbalance (hyperkalemia, hyperphosphatemia), and osteoporosis, which increase the medical and socioeconomic burden. Our study suggests that patients who develop T2DM at an early age may require intensive medical treatment and more-frequent screening for renal complications. In the subgroup analysis, a low BMI and non-smoking status were more important risk factors for CKD development in YOD. Among patients with YOD, hypertension or dyslipidemia, and the use of sulfonylurea were more common in those who developed CKD. Low-BMI and non-smoking populations are generally regarded as low-risk metabolic groups. Our data suggest that, although patients may have a low BMI or do not smoke, they should require more medical attention for renal complications if they develop T2DM at an early age. Our data also suggest that patients with YOD who have concomitant hypertension or dyslipidemia should be aware of the potential development of nephropathy^31^.

At this point, we do not fully understand the mechanism by which sulfonylurea use is associated with an increased incidence of CKD in YOD. Because sulfonylurea is prescribed commonly in Korea to patients who do not reach optimal glycemic targets despite using metformin and DPP-IV inhibitors, we speculate that a higher proportion of sulfonylurea usage may be related to hyperglycemia in patients with YOD. In addition, sulfonylurea-associated hypoglycemia may have contributed to the increased development of CKD^32^. However, because we could not measure the incidence of hypoglycemic events in our participants, we do not have data to verify our hypothesis.

Anti-diabetic agents, such as SGLT2 inhibitors (SGLT2i) or GLP1 receptor agonists (GLP1-RA), have been shown to delay the progression of CKD (microalbuminuria, GFR)^33–36^. Our study enrolled patients who were newly diagnosed diabetes during 2010 to 2011, when neither SGLT2i (Dapagliflozin, Empagliflozin, Canagliflozin) nor GLP1-RAs (Liraglutide, Dulaglutide, Semaglutide) with proven benefit in renal outcome were available in Korea. Even by the period of study termination, the prescription rate of SGLT2i was low (2% in 2015 and 3.2% in 2016)^37^. Therefore, we speculate the number of patients who used SGLT2i and GLP1-RA was very small in our study. Recently, Liraglutide, Dulaglutide, and Dapagliflozin have been shown to be safe, even in adolescents^38–40^. Studies evaluating whether patients with YOD may benefit from the early use of these anti-diabetic agents will be interesting subject to be explored.

Despite the increasing prevalence, the clinical course of YOD is poorly understood, particularly with respect to renal outcomes. Previous studies reported high comorbidity of nephropathy in patients with YOD. Patients with YOD have a higher incidence of renal complications than patients with Type 1 diabetes (T1DM) of similar age^41^. Compared with LOD, the prevalence of nephropathy is higher in YOD at any given age^42,43^. However, whether the risk of developing nephropathy is fundamentally different for patients with YOD compared to those with LOD remains inconclusive^7^. Pavkov et al. longitudinally analyzed 1856 Pima Indians and demonstrated that the incidence of ESRD did not differ between patients with early-onset diabetes and LOD^42^. However, the definition of early-onset diabetes in that study was different from that used in our study. In that study, patients who were diagnosed with T2DM under the age of 20 years were defined as having YOD, whereas patients diagnosed aged 20–55 years were defined as having LOD. Therefore, we suggest that the LOD group in that study might have included some patients who share the same clinical characteristics of YOD. Chan et al. studied 9509 Chinese patients and reported that, at any given age, patients with YOD are at an increased risk of developing CKD compared to patients with LOD^43^. The authors also showed that, when adjusted for the duration of diabetes, the incidence of CKD was even higher in patients with LOD than in those with YOD. However, their cohort included a significant portion of patients already diagnosed with diabetes at the time of enrollment (baseline mean duration of diabetes: YOD = 6 years, LOD = 5 years), with the baseline GFR being lower in patients with LOD (96.9 ± 32.0 mL/min/1.73 m^2^) than in patients with YOD (125.1 ± 36.7 mL/min/1.73 m^2^). Because the incidence of CKD was defined in this study as a GFR < 60 mL/min/1.73 m^2^, the possibility of overestimating the relative risk cannot be excluded. Recently, Wu et al. studied 436,744 newly diagnosed (Hong Kong Diabetes Surveillance Database, HKDSD) and 16,979 already diagnosed (Hong Kong Diabetes Register) T2DM patients in China. Consistent with our study, the authors maintained that the increased risk of CKD in YOD can be attributed to both increased exposed diabetic condition and aggressiveness of disease^44^.

A large number of subjects and homogeneity of ethnicity are two strengths of our study. We analyzed 83,032 (YOD = 7345) patients newly diagnosed with Type 2 diabetes to determine the risk of CKD development. Previous studies suggest that the heterogeneity of the incidence and clinical characteristics of YOD is dependent on ethnicity^7,45^. The participants in our study comprised patients in Korea only. Importantly, the baseline GFR was lower in the LOD group (91.33 ± 36.64 mL/min/1.73 m^2^) compared with the YOD group (98.92 ± 45.06 mL/min/1.73 m^2^) in our cohort. However, the risk of CKD was higher in patients with YOD than in those with LOD, even after adjusting for clinically distinct variables. Therefore, we speculate the relative CKD risk in the YOD group would not be overestimated in our study.

Here, the risk of developing CKD was consistently higher in patients with YOD than in those with LOD, even as clinically distinct variables were serially adjusted. In particular, a higher risk of CKD development in the YOD group was more prominent in the non-smoking or low-BMI subgroups. High BMI and smoking are well known risk factor for the development of CKD^46,47^. Our data suggests that the presence of YOD can be more critical factor for the development in CKD in this relatively CKD-low risk population (low-BMI or non-smoking). Although, we cannot exclude the possibility that the risk of CKD could have been overestimated in non-smoking or low-BMI group since we have adjusted smoking and BMI during our analysis. Our results suggest that patients with YOD may have different pathophysiological background from that of LOD; these could have contributed to the difference in CKD development. However, we could not measure important parameters, such as HbA1c or C-peptide levels, which may potentially affect the renal outcome. We also could not measure the variability of some metabolic parameters (fasting plasma glucose, blood pressure, and cholesterol, uric acid), which can also affect renal outcomes^48^.

We also could not measure potential renal affecting condition such as proton pump inhibitor, non-steroid anti-inflammatory drug or herbal use. Although we could not encompass every renal confounding factors due to data availability, we tried to overcome this limitation by defining CKD when GFR was measured < 60 mL/min/1.73 m^2^ consecutively twice or more (stage 3 or higher) during the health examination. Because National Health Surveillance program is performed every 2 years, we speculate most of the transient GFR decrease due to medication use could be excluded. As for the definition of CKD, although a similar definition has been used in previous studies to define diabetic nephropathy, we admit that patients with micro- or macro-albuminuria with GFR > 60 mL/min/1.73 m^2^ were potentially included in this study^43,49^. We defined the diagnosis of Type 2 diabetes as the time point when anti-diabetic medication was first prescribed to those who attained diabetes-related ICD-10 codes (E11–14) at least once per year during the observational period. For this reason, there could be a time difference between the actual T2DM onset and the time point defined by the T2DM diagnosis. However, since patients with LOD tend to start medication later than patients with YOD, we speculate that this technical barrier would not have biased our results. We enrolled patients newly diagnosed with T2DM by considering patients who started oral hypoglycemic agents and excluded patients with T1DM. Therefore, minor forms of diabetes, such as steroid-induced diabetes, pancreatitis, pancreatectomy-induced diabetes, monogenic diabetes, or latent autoimmune diabetes in adults could have been included in our study. However, considering the large number of patients analyzed (n = 84,384), we suggest that the inclusion of patients with minor forms of diabetes would not have biased the overall conclusion of our study.

In summary, among the Korean population, patients with YOD are at an increased risk of developing CKD compared with patients with LOD. The risk of developing CKD in patients with YOD is higher than that in patients with LOD, even after adjusting for clinically distinct characteristics that include age, sex, BMI, smoking, heavy drinking, regular physical activity, low income, hypertension, aspirin use, fasting glucose levels, insulin use, and the number of oral hypoglycemic agents. This suggests that for patients who develop T2DM at an early age more attention is required for the development and prevention of renal complications.

## Supplementary Information

Supplementary Table 1.

## Data Availability

The data that support the findings of this study are available from National Health information database from National Health Insurance Sharing Service (NHISS, https://nhiss.nhis.or.kr/bd/ab/bdaba000eng.do) but restrictions apply to the availability of these data, which were used under license for the current study, and so are not publicly available. Data are however available from the authors upon reasonable request and with permission of NHISS.

## References

[1] Shaw JE, Sicree RA, Zimmet PZ (2010). Global estimates of the prevalence of diabetes for 2010 and 2030. Diabetes Res. Clin. Pract..

[2] Telo GH, Cureau FV, Szklo M, Bloch KV, Schaan BD (2019). Prevalence of Type 2 diabetes among adolescents in Brazil: Findings from study of cardiovascular risk in adolescents (ERICA). Pediatr. Diabetes.

[3] FazeliFarsani S, van der Aa MP, van der Vorst MM, Knibbe CA, de Boer A (2013). Global trends in the incidence and prevalence of Type 2 diabetes in children and adolescents: A systematic review and evaluation of methodological approaches. Diabetologia.

[4] Magliano DJ, Boyko EJ (2021). IDF Diabetes Atlas in Idf Diabetes Atlas.

[5] Fu JF (2013). Status and trends of diabetes in Chinese children: Analysis of data from 14 medical centers. World J. Pediatr..

[6] Likitmaskul S (2003). Increasing prevalence of Type 2 diabetes mellitus in Thai children and adolescents associated with increasing prevalence of obesity. J. Pediatr. Endocrinol. Metab..

[7] Magliano DJ (2020). Young-onset Type 2 diabetes mellitus: Implications for morbidity and mortality. Nat. Rev. Endocrinol..

[8] Nanayakkara N (2018). Younger people with Type 2 diabetes have poorer self-care practices compared with older people: Results from the Australian National Diabetes Audit. Diabet. Med..

[9] Kaar JL (2020). Evaluation of the longitudinal change in health behavior profiles across treatment groups in the TODAY clinical trial. Pediatr. Diabetes.

[10] Kahn SE (2011). Effects of rosiglitazone, glyburide, and metformin on β-cell function and insulin sensitivity in ADOPT. Diabetes.

[11] Hillier TA, Pedula KL (2001). Characteristics of an adult population with newly diagnosed Type 2 diabetes: The relation of obesity and age of onset. Diabetes Care.

[12] Al-Saeed AH (2016). An inverse relationship between age of Type 2 diabetes onset and complication risk and mortality: The impact of youth-onset Type 2 diabetes. Diabetes Care.

[13] Huo L (2018). Impact of age at diagnosis and duration of Type 2 diabetes on mortality in Australia 1997–2011. Diabetologia.

[14] Hillier TA, Pedula KL (2003). Complications in young adults with early-onset Type 2 diabetes: Losing the relative protection of youth. Diabetes Care.

[15] Krakoff J (2003). Incidence of retinopathy and nephropathy in youth-onset compared with adult-onset Type 2 diabetes. Diabetes Care.

[16] Song SH, Gray TA (2011). Early-onset Type 2 diabetes: High risk for premature diabetic retinopathy. Diabetes Res. Clin. Pract..

[17] UK Prospective Diabetes Study Group (1998). Tight blood pressure control and risk of macrovascular and microvascular complications in Type 2 diabetes: UKPDS 38. BMJ.

[18] Zoungas S (2009). Combined effects of routine blood pressure lowering and intensive glucose control on macrovascular and microvascular outcomes in patients with Type 2 diabetes: New results from the ADVANCE trial. Diabetes Care.

[19] UK Prospective Diabetes Study Group (1998). Intensive blood-glucose control with sulphonylureas or insulin compared with conventional treatment and risk of complications in patients with Type 2 diabetes (UKPDS 33). Lancet.

[20] Lee YH, Han K, Ko SH, Ko KS, Lee KU (2016). Data analytic process of a nationwide population-based study using national health information database established by national health insurance service. Diabetes Metab. J..

[21] Kim MK, Han K, Lee SH (2022). Current trends of big data research using the Korean national health information database. Diabetes Metab. J..

[22] Eppens MC (2006). Prevalence of diabetes complications in adolescents with type 2 compared with type 1 diabetes. Diabetes Care.

[23] Constantino MI (2013). Long-term complications and mortality in young-onset diabetes: Type 2 diabetes is more hazardous and lethal than type 1 diabetes. Diabetes Care.

[24] Musso CG, Oreopoulos DG (2011). Aging and physiological changes of the kidneys including changes in glomerular filtration rate. Nephron Physiol..

[25] Stevens LA, Viswanathan G, Weiner DE (2010). Chronic kidney disease and end-stage renal disease in the elderly population: Current prevalence, future projections, and clinical significance. Adv. Chronic Kidney Dis..

[26] Seo MH (2019). 2018 Korean society for the study of obesity guideline for the management of obesity in Korea. J. Obes. Metab. Syndr..

[27] Lee M-K, Han K, Kwon H-S (2019). Age-specific diabetes risk by the number of metabolic syndrome components: A Korean nationwide cohort study. Diabetol. Metab. Syndr..

[28] Consortium T. R. (2019). Effects of treatment of impaired glucose tolerance or recently diagnosed Type 2 diabetes with metformin alone or in combination with insulin glargine on β-cell function: Comparison of responses in youth and adults. Diabetes.

[29] Reynolds K (2018). Mortality in youth-onset type 1 and Type 2 diabetes: The SEARCH for diabetes in youth study. J. Diabetes Compl..

[30] Mast R (2016). Time to insulin initiation and long-term effects of initiating insulin in people with Type 2 diabetes mellitus: The Hoorn diabetes care system cohort study. Eur. J. Endocrinol..

[31] Nissenson AR (2014). Improving outcomes for ESRD patients: Shifting the quality paradigm. Clin. J. Am. Soc. Nephrol..

[32] Yun J-S (2021). Severe hypoglycemia and the risk of end stage renal disease in Type 2 diabetes. Sci. Rep..

[33] Gerstein HC (2019). Dulaglutide and renal outcomes in Type 2 diabetes: An exploratory analysis of the REWIND randomised, placebo-controlled trial. Lancet.

[34] Heerspink HJL (2020). Dapagliflozin in patients with chronic kidney disease. N. Engl. J. Med..

[35] Mann JFE (2017). Liraglutide and renal outcomes in Type 2 diabetes. N. Engl. J. Med..

[36] Wanner C (2016). Empagliflozin and progression of kidney disease in Type 2 diabetes. N. Engl. J. Med..

[37] Baek JH (2022). Real-world prescription patterns and barriers related to the use of sodium-glucose cotransporter 2 inhibitors among korean patients with Type 2 diabetes mellitus and cardiovascular disease. Diabetes Metab. J..

[38] Kelly AS (2020). A randomized, controlled trial of liraglutide for adolescents with obesity. N. Engl. J. Med..

[39] Arslanian SA (2022). Once-weekly dulaglutide for the treatment of youths with Type 2 diabetes. N. Engl. J. Med..

[40] Tamborlane WV (2022). Efficacy and safety of dapagliflozin in children and young adults with Type 2 diabetes: A prospective, multicentre, randomised, parallel group, phase 3 study. Lancet Diabet. Endocrinol..

[41] Dabelea D (2017). Association of type 1 diabetes vs Type 2 diabetes diagnosed during childhood and adolescence with complications during teenage years and young adulthood. JAMA.

[42] Pavkov ME (2006). Effect of youth-onset Type 2 diabetes mellitus on incidence of end-stage renal disease and mortality in young and middle-aged Pima Indians. JAMA.

[43] Chan JC (2014). Premature mortality and comorbidities in young-onset diabetes: A 7-year prospective analysis. Am. J. Med..

[44] Wu H (2021). Young age at diabetes diagnosis amplifies the effect of diabetes duration on risk of chronic kidney disease: A prospective cohort study. Diabetologia.

[45] Yang F, Han YSF, Sohn KF, Kim TSF, Young NH (2021). onset Type 2 diabetes in South Korea: A review of the current status and unmet need. Korean J. Intern. Med..

[46] Hsu CY, McCulloch CE, Iribarren C, Darbinian J, Go AS (2006). Body mass index and risk for end-stage renal disease. Ann. Intern. Med..

[47] Yacoub R (2010). Association between smoking and chronic kidney disease: A case control study. BMC Public Health.

[48] Kim MK (2019). Effects of variability in blood pressure, glucose, and cholesterol concentrations, and body mass index on end-stage renal disease in the general population of Korea. J. Clin. Med..

[49] Foundation NK (2002). K/DOQI clinical practice guidelines for chronic kidney disease: Evaluation, classification, and stratification. Am. J. Kidney Dis..

