# Supplementary material for: Risk of developing chronic kidney disease in young-onset Type 2 diabetes in Korea
Source: Sci Rep. 2023 Jun 21;13:10100. doi: 10.1038/s41598-023-36711-2 (PMC10284790; doi:10.1038/s41598-023-36711-2)
Supplement: Supplementary file 1 — Supplementary Table 1. [file 41598_2023_36711_MOESM1_ESM.docx]

**Supplementary Table 1. Baseline characteristics of patients who developed or non-developed CKD during the follow-up period**

|  | **Total subjects** | | | | |  | **YOD** | | |
| --- | --- | --- | --- | --- | --- | --- | --- | --- | --- |
|  | **CKD**  **non-developed** | **CKD**  **developed** | **p-value** |  | **CKD**  **non-developed** | | | **CKD**  **developed** | **p-value** |
| **Number, n (%)** | 82,942 | 1442 |  |  | 7,311 | | | 34 |  |
| **Sex: Male, n (%)** | 54,001 (65.11) | 965 (66.92) | 0.1518 |  | 5,963 (81.56) | | | 30 (88.24) | 0.3165 |
| **Age** | 51.27 ± 8.07 | 56.25 ± 6.48 | <.0001 |  | 35.05 ± 3.65 | | | 36.44 ± 2.62 | 0.0268 |
| **Height (cm)** | 164.43 ± 8.68 | 163.53 ± 8.52 | <.0001 |  | 170.58 ± 7.69 | | | 171.47 ± 7.72 | 0.499 |
| **Weight (kg)** | 69.45 ± 11.84 | 69.2 ± 11.04 | 0.4208 |  | 78.77 ± 14.44 | | | 79.35 ± 17.28 | 0.8147 |
| **BMI (kg/m^2^)** | 25.6 ± 3.33 | 25.82 ± 3.2 | 0.0155 |  | 26.98 ± 4.16 | | | 26.79 ± 4.53 | 0.7901 |
| **Obesity (BMI ≥ 25 kg/m^2^), n (%)** | 44,991 (54.24) | 840 (58.25) | 0.0024 |  | 4,955 (67.77) | | | 19 (55.88) | 0.139 |
| **Waist circumference (cm)** | 85.85 ± 8.51 | 87 ± 8.12 | <.0001 |  | 88.15 ± 10.14 | | | 89.38 ± 11.82 | 0.4814 |
| **Abdominal Obesity, n (%)** | 65039 (78.42) | 1194 (82.8) | <.0001 |  | 5,978 (81.77) | | | 28 (82.35) | 0.9297 |
| **Smoking, n (%)** | 23685 (28.56) | 372 (25.8) | 0.0214 |  | 3,353 (45.86) | | | 11 (32.35) | 0.1147 |
| **Heavy drinker, n (%)** | 9132 (11.01) | 127 (8.81) | 0.008 |  | 944 (12.91) | | | 3 (8.82) | 0.4779 |
| **Regular physical activity, n (%)** | 17575 (21.19) | 322 (22.33) | 0.2935 |  | 1,233 (16.86) | | | 5 (14.71) | 0.7372 |
| **Low socioeconomic status, n (%)** | 17941(21.63) | 402 (27.88) | <.0001 |  | 954 (13.05) | | | 7 (20.59) | 0.1934 |
| **Hypertension, n (%)** | 43348 (52.26) | 1100 (76.28) | <.0001 |  | 2,541 (34.76) | | | 22 (64.71) | 0.0003 |
| **Dyslipidemia, n (%)** | 42,710 (51.49) | 886 (61.44) | <.0001 |  | 3,427 (46.87) | | | 25 (73.53) | 0.0019 |
| **Medication, n (%)** |  |  |  |  |  | | |  |  |
| **Aspirin** | 21,658 (26.11) | 616 (42.72) | <.0001 |  | 748 (10.23) | | | 5 (14.71) | 0.3908 |
| **Statin** | 38,905 (46.91) | 850 (58.95) | <.0001 |  | 2,761 (37.77) | | | 23 (67.65) | 0.0003 |
| **ARB** | 30,575 (36.86) | 875 (60.68) | <.0001 |  | 1,776 (24.29) | | | 19 (55.88) | <.0001 |
| **Insulin, n (%)** | 5929 (7.15) | 119 (8.25) | 0.1071 |  | 886 (12.12) | | | 5 (14.71) | 0.6448 |
| **Oral hypopglycemic agents, n (%)** |  |  |  |  |  | | |  |  |
| **Sulfonylurea** | 27,985 (33.74) | 519 (35.99) | 0.0731 |  | 2,598 (35.54) | | | 18 (52.94) | 0.0345 |
| **Metformin** | 65,454 (78.92) | 1089 (75.52) | 0.0017 |  | 5,880 (80.43) | | | 23 (67.65) | 0.0613 |
| **Meglitinide** | 718 (0.87) | 24 (1.66) | 0.0013 |  | 64 (0.88) | | | 0 (0) | 0.5837 |
| **Thiazolidinedione** | 2,395 (2.89) | 42 (2.91) | 0.9551 |  | 226 (3.09) | | | 2 (5.88) | 0.3492 |
| **DPP-4 inhibitor** | 7,163 (8.64) | 102 (7.07) | 0.036 |  | 779 (10.66) | | | 4 (11.76) | 0.8343 |
| **α-glucosidase inhibitor** | 2,986 (3.6) | 61 (4.23) | 0.2035 |  | 286 (3.91) | | | 2 (5.88) | 0.5548 |
| **Number of oral hypoglycemic agents** |  |  | 0.4887 |  |  | | |  | 0.4653 |
| **1** | 56,071 (67.6) | 984 (68.24) |  |  | 4,277 (58.5) | | | 18 (52.94) |  |
| **2** | 2,4287 (29.28) | 407 (28.22) |  |  | 2,700 (36.93) | | | 13 (38.24) |  |
| **≥ 3** | 2,584 (3.12) | 51 (3.54) |  |  | 334 (4.57) | | | 3 (8.82) |  |
| **Number of oral hypoglycemic agents** | 1.36 ± 0.55 | 1.36 ± 0.56 | 0.9165 |  | 1.47 ± 0.6 | | | 1.59 ± 0.74 | 0.2396 |
| **Fasting glucose (mg/dl)** | 144.69 ± 51.09 | 145.11 ± 55.84 | 0.7618 |  | 158.75 ± 65.74 | | | 167.62 ± 90.51 | 0.4338 |
| **Systolic BP (mmHg)** | 127.14 ± 14.78 | 130.05 ± 15.78 | <.0001 |  | 125.87 ± 13.98 | | | 129.18 ± 19.26 | 0.1693 |
| **Diastolic BP (mmHg)** | 79.72 ± 9.97 | 80.87 ± 10.59 | <.0001 |  | 79.83 ± 10.15 | | | 81.85 ± 13.45 | 0.2482 |
| **Total Cholesterol (mg/dl)** | 203.66 ± 42.23 | 202.89 ± 46.66 | 0.496 |  | 205.54 ± 42.62 | | | 206.85 ± 53.31 | 0.8578 |
| **Triglyceride (mg/dl)** | 182.54 ± 116.12 | 198.25 ± 126.24 | <.0001 |  | 216.07 ± 145.59 | | | 251.09 ± 204.84 | 0.1627 |
| **HDL-C (mg/dl)** | 50.96 ± 14.56 | 50.62 ± 18.04 | 0.3803 |  | 48.58 ± 15.32 | | | 48.94 ± 14.15 | 0.8908 |
| **LDL-C (mg/dl)** | 117.4 ± 46.25 | 113.1 ± 41.76 | 0.0005 |  | 116.42 ± 53.44 | | | 108.35 ± 46.94 | 0.3796 |
| **GFR (ml/min/1.73 m^2^)** | 92.23 ± 37.57 | 78.35 ± 30.94 | <.0001 |  | 98.99 ± 45.14 | | | 84.65 ± 18.34 | 0.0642 |

ARB, angiotensin II receptor blocker; BMI, body mass index; BP, blood pressure; CKD, chronic kidney disease; GFR, glomerular filtration rate; HDL-C, high-density lipoprotein cholesterol; LDL-C, low-density lipoprotein cholesterol
